# Determinants of modern contraceptive use among postpartum women in two health facilities in urban Ghana: a cross-sectional study

**DOI:** 10.1186/s40834-019-0098-9

**Published:** 2019-10-21

**Authors:** Jonathan Ian Coomson, Abubakar Manu

**Affiliations:** 1 3M&C Health System, LG DTD 10081, East Legon, Accra, Ghana; 2 Department of Population, Family and Reproductive Health, School of Public Health, University of Ghana, Accra, Ghana

**Keywords:** Modern contraceptives, Postpartum, Family planning, Women, Ghana

## Abstract

**Background:**

Postpartum contraception is important for spacing and limiting childbirth. Although the use of modern contraception has been shown to reduce maternal and child morbidities and mortalities, postpartum women have one of the highest unmet needs for family planning. Inter-birth intervals less than 24 months have adverse effects on both the mother and the child, yet very limited empirical evidence exist on contraceptive use among postpartum women in Ghana. This study sought to determine the prevalence and determinants of modern contraceptive use among postpartum women in the Tema Metropolis, Ghana.

**Methods:**

A facility-based cross-sectional survey was conducted among 320 postpartum women with babies aged between three and 15 months. Participants were recruited from child welfare clinics in two government health facilities in the Tema metropolitan area using a simple random sampling technique. Data were analyzed using STATA version 15. Chi-square and multiple logistic regressions techniques were used to examine associations between postpartum contraceptive use and key independent variables. Statistical significance was set at *p* = 0.05. Adjusted odds ratios and their 95% confidence intervals were used to assess the strength of association.

**Results:**

The prevalence rate of modern contraceptive use among postpartum women was 26.3%. Postpartum contraceptive use was significantly associated with past contraceptive use [AOR = 7.7 (95%CI: 3.4–17.5)]; return of menses [AOR = 4.3 (95%CI: 1.7–11.3)]; resumption of sexual activity [AOR = 4.7 (95%CI: 1.4–15.4)]; discussion of family planning with male partner [AOR = 3.1 (95%CI: 1.03–9.2)]; male partners’ approval of modern contraception [AOR = 18.1 (95%CI: 6.3–51.6)]; family planning counselling received during antenatal care [AOR = 3.5 (95%CI: 1.3–9.9)] and knowledge of at least one modern methods of contraception available at the health facility [AOR = 4.7 (95%CI: 1.9–11.5)].

**Conclusions:**

Postpartum contraceptive uptake is low among women in the Tema area. Factors that influence modern contraceptive uptake among postpartum women include past modern contraceptive use, resumption of sexual activity and menstruation, male partner involvement in contraception, family planning counselling during antenatal care and knowledge of the modern methods of contraception available at the health facility. Strengthening family planning education and counselling during antenatal care and using a multi-prong strategy to engage men as partners in family planning will improve postpartum contraceptive uptake.

## Background

Postpartum contraception is one of the means to prevent rapid repeat pregnancies to ensure good health outcomes in both mother and baby. However, in developing countries, postpartum contraceptive uptake is low [1]. The World Health Organization estimates that about 830 women die daily from complications of pregnancy and childbirth worldwide, and nearly all (99%) of these maternal deaths occur in developing countries [2]. An overwhelming number of these deaths could be prevented through interventions such as the use of modern contraceptive methods. Evidence shows that an estimated 20% of obstetric deaths would be prevented if modern contraceptive methods were used [3].

Postpartum women have one of the greatest unmet need for family planning but most often than not, do not receive the services needed in ensuring longer birth intervals and reducing unintended pregnancies [4]. In Sub-Saharan Africa (SSA), the low use of modern contraceptives has led to high rates of unintended pregnancies, unsafe abortions and unplanned births [5]. About two-thirds of women would like to avoid pregnancy in the first year after delivery but are not on any postpartum contraceptive method [6, 7]. Evidence shows that nearly 95% of women who are 0 to 12 months postpartum desire to avoid pregnancy in the next 24 months, but 70% of them do not use contraception [8]. Short inter-birth intervals, that is, inter-birth intervals less than 24 months, are associated with maternal morbidities such as uterine rupture and uteroplacental bleeding disorders (abruptio placenta and placenta praevia) and infant morbidities such as prematurity, low birth weight and stunting [9]. Research indicates that in developing countries, the death rate of children under 5 years would reduce by 13% if women waited for at least 24 months after birth before conceiving, while a 25% decrease would be achieved if the waiting time was at least 36 months [10].

Although knowledge of contraceptives is relatively high among women of reproductive age in Ghana, its use remains low [11]. In Ghana, contraceptive prevalence rate among married women is only 27% for all methods, and 22% for modern methods [12]. Some studies have looked at the predictors of contraceptive use by postpartum women in developing countries of which Ghana is included. However, most of these studies used secondary data from the Ghana Demographic and Health Survey (GDHS) in 2008. Published research materials on postpartum contraceptives concerning Ghana are mostly those done on the global or regional scale using secondary data from demographic and health surveys [1, 13, 14]. Recently published research works which used primary data to study the factors that influence postpartum contraceptive use in Ghana are limited. This study, therefore, seeks to estimate the prevalence of postpartum modern contraceptive use and to assess the factors that influence modern contraceptive use among postpartum women in the Tema area of Ghana. In-depth knowledge of the factors that influence the use of postpartum modern contraceptives could be used to plan future educational programs on postpartum contraceptive, thereby reducing the maternal and child morbidities and mortalities associated with short inter-birth intervals. Results of this study could also be used for institutional assessment and implementing reforms at the facility level to increase the uptake of postpartum modern contraceptives.

## Methods

### Study design

This study employed a facility-based cross-sectional design using the quantitative method with structured questionnaires to obtain data from postpartum women with babies aged between three and 15 months.

### Study area, population and period

The study was conducted from May to June 2018, at the Tema General Hospital (TGH) and the Tema Polyclinic (TP). The facilities, located in the Tema Metropolis, about 30 km to the East of Accra, are the two main government facilities in the Tema District, both of which are operated under the Ghana Health Service (GHS) and offer various services including reproductive and child health services. The study population was all postpartum women attending the child welfare clinic of TGH and TP.

### Sample size determination and sampling procedure

The sample size was calculated based on Cochran’s formula n ≥ [$\left({Z}_{\frac{\propto }{2}}\right)$ ^2^ P (1-P)]/d^2^.

[15], with the following assumptions: Prevalence (P) of postpartum modern contraceptive use = 22.6% [16], margin of error (d) = 5%, ${Z}_{\frac{\propto }{2}}$ = 1.96 at 95% confidence interval and a non-response rate of 10%. The total minimum sample size was 300 participants.

Proportion-to-size sampling was done to know the number of participants that was needed from each facility. Based on average monthly attendance at the two child welfare clinics, about 68% of the participants were recruited from TGH while the remaining 32% were recruited from TP.

At the facility level, a simple random sampling technique was used in selecting participants in the study. Postpartum women assessing the child welfare clinic of TGH and TP who met the criteria for the study were numbered from one till the last person. Pieces of papers with the corresponding numbers just like the numbering of the women were put in an opaque container, mixed thoroughly and blindly selected by a volunteer midwife. Postpartum women who had their numbers selected were then invited to participate in the study for that particular day after informed consent had been sought from them. With the help of four research assistants, a maximum of 25 participants were interviewed per day in TGH whilst that of TP was a maximum of 10 interviews per day.

### Inclusion criteria

1. All women of reproductive age who delivered 3–15 months prior to the study, accessing the child welfare clinic of TGH and TP and who were willing to be participants in the study.
2. For postpartum women who were below 18 years at the time of data collection, written informed consent were sought from their guardian or parent who accompanied them to the hospital. Postpartum mothers below the age of 18 years who came to the health facility without a guardian or parent were excluded from the study.

### Exclusion criteria

1. Postpartum women who had had a hysterectomy (surgical removal of the uterus).
2. Postpartum women who did not speak and understand English, Twi or Ga were excluded from the study due to the language barrier.
3. Postpartum women who were pregnant at the time of accessing the Child Welfare Clinic of TGH and TP.

### Measures

Postpartum modern contraceptive use was the dependent variable; and was measured directly with a yes/no response (yes = 1 and no = 0). Postpartum women were asked to indicate whether they were currently using a modern contraceptive method.

The independent variables such as the use of modern contraceptives before last delivery, resumption of sexual activity, resumption of menstruation after delivery, discussion of family planning with male partner after delivery of last child, male partner’s approval of modern contraceptives, antenatal and postnatal care attendance and whether family planning counselling occurred during these visits were measured directly with yes or no questions where yes = 1 and no = 0.

Regarding knowledge of modern contraceptive methods available at the health facility, women were asked a yes/no question on whether they knew the available contraceptive methods being offered at the health facility. Women who responded in the affirmative were asked to name the types of contraceptives being offered at the health facility. Women who correctly named at least one method were deemed to have knowledge of the available methods at the health facility. Women who said they knew the available methods but could not mention correctly any method as well as those who answered no were assumed to have no knowledge of the contraceptive methods available at the facility.

Knowledge of postpartum modern contraceptive side effects was computed by asking women to list the side effects of modern contraceptives they knew. Women who knew no side effect or correctly listed only one side effect were assumed to have a low knowledge of contraceptive side effects. Participants who correctly listed between two to three side effects were deemed to have moderate knowledge. Postpartum women who knew four or more side effects of modern contraceptives were assumed to have high knowledge of the side effects of modern contraceptives.

Knowledge of postpartum physiology was a created composite variable, where women were asked three yes/no questions where yes = 1 and no = 0. The questions included whether or not a postpartum woman can become pregnant when breastfeeding, can become pregnant when her menses has not returned and whether sexual activity had any effect on the time of the return of her menstrual cycle after delivery. A score of 0 or 1 was deemed as low knowledge, 2 as moderate knowledge and 3 as high knowledge.

### Data quality assurance

Four research assistants were recruited and trained. They were trained to ensure correct interpretation of the questionnaires. Research assistants were also trained on how to protect the confidentiality and privacy of participants. The principal investigator supervised and monitored the activities of the research assistants daily during data collection. Questionnaires were constructed in a lay-man’s language so as to make it easily understandable and precise as much as possible after a pre-test was conducted on 30 individuals at both the Lekma Hospital and Polyclinic.

### Data processing and analysis

Data were entered into Microsoft Excel spreadsheet (Microsoft Office 2010) and cleaned to eliminate all irregularities. The data were then exported into STATA version 15, coded and cleaned for analysis. Univariate analysis was carried out using frequencies and percentages to describe the exposure and outcome variables independently. Bivariate analysis using the Pearson’s Chi square was carried out to examine associations between postpartum modern contraceptive use and the various independent variables. All the independent variables which were found to be significantly associated with postpartum modern contraceptive use were fitted in a multivariate analysis using the binary logistic regression technique to determine predictors of postpartum contraceptive use. Odds ratios and their 95% confidence intervals were used to assess the strength of association. A *p*-value of 0.05 was used to determine statistical significance.

## Results

### Socio-demographic characteristics of respondents

Table 1 presents socio-demographic characteristics of study participants. The median age of participants was 29 years (IQR: 26–33.5). About half of the respondents 159 (49.7%) were between the ages of 20–29 years. Participants’ age ranged between 17 and 47 years. Majority of the study participants 284 (88.7%) were married or cohabiting. Only 14 (4.4%) of respondents had no formal education with a vast majority 280 (87.6%) having at least secondary or vocational education. Nearly a quarter 77 (24.1%) of respondents were unemployed. Almost all women in the study (97.2%) had partners who had at least primary education.

**Table 1:** Socio-demographic characteristics of respondents

Characteristics	Frequency (*n* = 320)	Percent
Age of mother		
≤ 19	9	2.8
20–29	159	49.7
30–39	137	42.8
40–49	15	4.7
Marital status		
Single/divorced/widowed	36	11.3
Married/cohabiting	284	88.7
Education of mother		
No formal education	14	4.4
Primary	26	8.1
Secondary/vocational	204	63.8
Tertiary	76	23.8
Occupation of mother		
Public salaried worker	32	10.0
Private salaried worker	66	20.6
Trader	113	35.3
Unemployed	77	24.1
Other	32	10.0
Education of partner		
No formal education	9	2.8
Primary	3	0.9
Secondary/vocational	183	57.2
Tertiary	125	39.1

### Reproductive health characteristics of respondents

The median number of pregnancies of respondents was 2.5 (IQR: 2–3). About two out of every five women (42.8%) had only one child. Modal age group of the last child of participants was 6–8 months. The median age of the last child was 7 months [5–9]. Almost two thirds (63.7%) of respondents had the desire to have another child and of those who desired to have another child, about 84% (171) of them desired to have the next child after 21 months from the time of the study. About two in five (43%) of participants said the pregnancy which resulted in the birth of their last child was not planned or intended. Majority of participants 315 (98.4) had their last delivery in a health facility. Almost all the women 315 (97.5%) had at least one antenatal visit with nearly half (46.5%) meeting the current WHO’s recommendation of at least eight antenatal care contacts before delivery [17]. Postnatal care attendance was almost universal 319 (99.7%) with nine in 10 women (90.9%) satisfying the WHO’s recommendation of at least three visits [18] during the postnatal care period (Table 2).

**Table 2:** Reproductive health characteristics of participants

Variable	Frequency (n = 320)	Percent
Number of past pregnancies		
1	64	20.0
2	96	30.0
3	88	27.5
4	54	16.8
More than 4	18	5.7
Number of children		
1	137	42.8
2	101	31.6
3	50	15.6
4	26	8.1
More than 4	6	1.9
Age of child		
3–5 months	98	30.6
6–8 months	111	34.7
9–11 months	75	23.4
12–15 months	36	11.3
Desires to have another child		
No	116	36.3
Yes	204	63.7
When next child is wanted	*N* = 204	
Within 21 months	33	16.2
After 21 months	171	83.8
Ever used modern contraceptive		
No	177	55.3
Yes	143	44.7
Last pregnancy planned/intended		
No	138	43.1
Yes	183	56.9
Menstruated after delivery		
No	124	38.7
Yes	196	61.3
Resumed sexual activity		
No	110	34.4
Yes	210	65.6
Current modern contraceptive use		
No	236	73.7
Yes	84	26.3
Discussed family planning with partner		
No	156	48.8
Yes	164	51.2
Partners’ approval of modern contraception		
No	101	31.6
Yes	111	34.7
I do not know	108	33.7
Place of delivery		
Health facility	315	98.4
home	5	1.6
Antenatal attendance		
No	8	2.5
Yes	312	97.5
Number of antenatal attendance	*N* = 312	
Less than 8 visits	167	53.5
8 or more visits	145	46.5
FP counselling at antenatal	*N* = 312	
No	80	25.6
Yes	232	74.4
Postnatal attendance		
No	1	0.3
Yes	319	99.7
Number of postnatal attendance	*N* = 319	
Less than 3 visits	29	9.1
3 or more visits	290	90.9
FP counselling at postnatal	*N* = 319	
No	200	62.7
Yes	119	37.3
Knowledge of side effects		
Low knowledge	108	33.8
Moderate knowledge	155	48.4
High knowledge	57	17.8
Knowledge of postpartum physiology		
Low knowledge	54	16.9
Moderate knowledge	131	41
High knowledge	135	42.1
Knowledge of FP methods available at health facility		
No	184	57.5
Yes	136	42.5

### Postpartum contraceptive prevalence rate

**Fig. 1:**
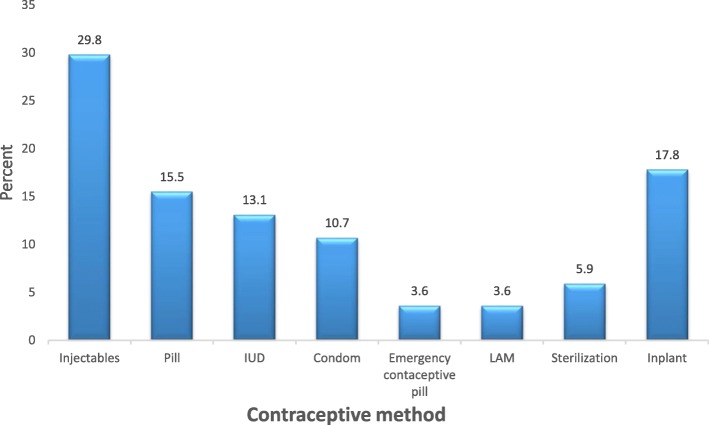
Preferred methods of postpartum modern contraception

**Fig. 2:**
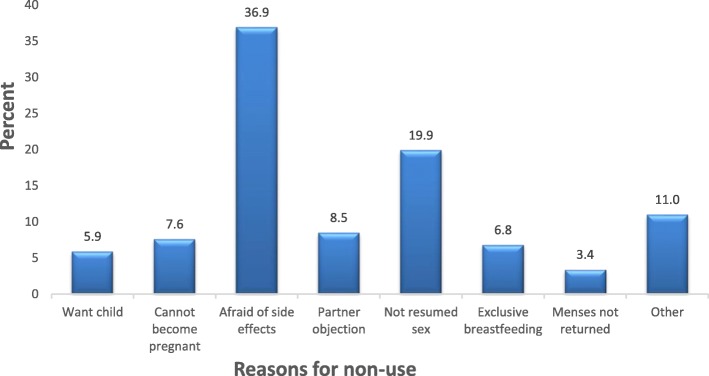
Reasons for non-use of postpartum modern contraception

Modern contraceptive prevalence among the postpartum women was 26.3 95% CI: 21.5–31.4%. Majority 177 (55.3%) of participants had never used any form of modern contraceptive method in their reproductive lives. Of the 143 participants who had used modern contraceptives in the past, a little over two-fifth (42.7%) obtained information about contraceptive use from health workers. Almost a third (29.8%) of postpartum women who were using modern contraceptives were using injectables (Fig. 1). Of the 284 (73.7%) postpartum women who were currently not using any modern method of contraception, more than one-third (36.9%) reported that fear of the side effects was the main problem for non-use of contraception (Fig. 2).

### Predictors of postpartum modern contraceptive use

Tables 3 and 4 summarize bivariate association between postpartum modern contraceptive use and socio-demographic and reproductive health characteristics respectively. Women’s age (*p* = 0.027) was the only socio-demographic characteristic found to be associated with postpartum modern contraceptive use. The use of modern contraceptive in the past (*p* < 0.001), return of menstruation (*p* = 0.049), resumption of sexual activity (p < 0.001) and discussion of family planning among spouses (*p* < 0.001) were significantly associated with current modern contraceptive use postpartum women. Other variables significantly associated with modern contraceptive use were male partner approval of modern contraception (*p* < 0.001), whether family planning counselling was received during antenatal care (*p* = 0.008), family planning counselling at postnatal care (p < 0.001) and knowing at least one modern contraceptive methods available at the health facility (p < 0.001).

**Table 3:** Bivariate analysis of socio-demographic characteristics associated with current postpartum modern contraceptive use

Characteristic	Contraceptive use, n (%)		Chi square	P-value
	No	Yes		
Age of mother				
10–19	**7 (**3.0)	2 (2.4)	9.20	0.027
20–29	126 (53.4)	33 (39.3)		
30–39	96 (40.6)	41 (48.8)		
40–49	7 (3.0)	8 (9.5)		
Marital status				
Single/widow/divorced	24 (10.2)	12 (14.3)	1.05	0.305
Married/cohabiting	212 (89.8)	72 (85.7)		
Education of mother				
No formal education	10 (4.3)	4 (4.8)		0.298*
Primary	22 (9.3)	4 (4.8)		
Secondary/vocational	144 (61.0)	60 (71.4)		
Tertiary	60 (25.4)	16 (19.0)		
Occupation of mother				
Unemployed	59 (25.0)	18 (21.4)	0.43	0.511
Employed	177 (75.0)	66 (78.6)		
Education of partner				
No formal education	8 (3.4)	1 (1.2)		0.349*
Primary	2 (0.9)	1 (1.2)		
Secondary/vocational	129 (54.6)	54 (64.3)		
Tertiary	97 (41.1)	28 (33.3)		

*Fisher’s exact

**Table 4:** Bivariate analysis of other factors associated with postpartum modern contraceptive use

Characteristic	Contraceptive use, n (%)		Chi square	p-value
	No	Yes		
Number of past pregnancies				
1	53 (22.5)	11 (13.1)	7.87	0.096
2	75 (31.8)	21 (25.0)		
3	62 (26.3)	26 (31.0)		
4	35 (14.8)	19 (22.6)		
More than 4	11 (4.6)	7 (8.3)		
Number of children				
1	107 (45.3)	30 (35.7)	7.39	0.117
2	72 (30.5)	29 (34.5)		
3	38 (16.1)	12 (14.3)		
4	14 (5.9)	12 (14.3)		
More than 4	5 (2.2)	1 (1.2)		
Age of child				
3–5 months	76 (32.2)	22 (26.2)	2.04	0.564
6–8 months	82 (34.8)	29 (34.5)		
9–11 months	51 (21.6)	24 (28.6)		
12–15 months	27 (11.4)	9 (10.7)		
Desires another child				
No	84 (14.5)	32 (21.2)	0.17	0.682
Yes	152 (85.5)	52 (78.8)		
When next child is desired				
Within 21 months	22 (66.7)	11 (33.3)	1.28	0.259
After 21 months	130 (76.0)	41 (24.0)		
Ever used modern contraceptive				
No	154 (65.3)	23 (27.4)	35.95	< 0.001
Yes	82 (34.7)	61 (72.6)		
Last pregnancy planned/intended				
No	101 (42.8)	37 (44.0)	0.04	0.842
Yes	135 (57.2)	47 (56.0)		
Menses returned				
No	99 (42.0)	25 (29.8)	3.88	0.049
Yes	137 (58.0)	59 (70.2)		
Resumed sexual activity				
No	103 (43.6)	7 (8.3)	34.24	< 0.001
Yes	133 (56.4)	77 (91.7)		
Discussed FP with partner				
No	144 (61.0)	12 (14.3)	54.15	< 0.001
Yes	92 (39.0)	72 (85.7)		
Partner approves modern contraception				
No	84 (35.6)	17 (20.2)	75.76	< 0.001
Yes	50 (21.2)	61 (72.6)		
I do not know	102 (43.2)	6 (7.2)		
Place of delivery				
Health facility	232 (98.3)	83 (98.8)		1.000*
home	4 (1.7)	1 (1.2)		
ANC attendance				
No	6 (2.5)	2 (2.4)		1.000*
yes	230 (97.5)	82 (97.6)		
FP counselling at antenatal				
No	68 (29.6)	12 (14.6)	7.068	0.008
Yes	162 (70.4)	70 (85.4)		
PNC attendance				
No	0 (0.0)	1 (1.2)		0.262*
Yes	236 (100.0)	83 (98.8)		
FP counselling at PNC				
No	166 (70.3)	34 (41.0)	22.66	< 0.001
Yes	70 (29.7)	49 (59.0)		
Knowledge of side effects				
Low knowledge	75 (31.8)	33 (39.3)	3.84	0.147
Moderate knowledge	122 (51.7)	33 (39.3)		
High knowledge	39 (16.5)	18 (21.4)		
Knowledge of postpartum physiology				
Low knowledge	41 (17.4)	13 (15.5)	1.42	0.491
Moderate knowledge	92 (39.0)	39 (46.4)		
High knowledge	103 (43.6)	32 (38.1)		
Knowledge of available FP methods				
No	163 (69.1)	21 (25.0)	49.23	< 0.001
Yes	73 (30.9)	63 (75.0)		

*Fishers’ exact

### Multivariate logistic regression

Table 5 presents results of multiple logistic regression of postpartum contraceptive use and selected independent variables. Postpartum women who had ever used a modern contraceptive were 7.7 times likely to be currently using a modern contraceptive compared to those who had never used a modern contraceptive method [AOR = 7.7 (95% CI: 3.4–17.5)]. Postpartum women whose menses had returned compared to those who were yet to have a return of menstrual flow were 4.3 times likely to be using a modern contraceptive method [AOR = 4.3 (95% CI: 1.7–11.3)]. Likewise, women who have resumed sexual activity have 4.7 times increased odds of using a modern method of contraception compared to those who are yet to resume sexual activity after delivery [AOR = 4.7 (95% CI: 1.4–15.4)]. Spouses who have discussed family planning have 310% increased odds of using a postpartum modern contraceptive compared to spouses who have not had any discussion on family planning [AOR = 3.1 (95% CI: 1.03–9.2)]. There was an 18.1 higher odds of using modern contraceptives among postpartum women whose partners’ approved of the use of modern contraceptives compared to women whose partners’ did not [AOR = 18.1 (95% CI: 6.3–51.6)]. Postpartum women who received family planning counselling during antenatal care visits at health facilities were 3.5 times likely to be on modern contraceptives during the study than those who said they received no family planning counselling during antenatal care [AOR = 3.5 (95% CI: 1.3–9.9)]. Lastly, postpartum women who knew at least a modern contraceptive method available at the health facility (Tema General Hospital and Tema Polyclinic) were 4.7 times likely to be currently using a modern method of contraception than women who had no idea of the contraceptive methods available at the health facility [AOR = 4.7 (95% CI: 1.9–11.5)].

**Table 5:** Logistic regression analysis of factors influencing postpartum modern contraceptive use

Variable	COR (95% CI)	p-value	AOR (95% CI)	p-value
Age of mother				
10–19	Ref		Ref	
20–29	0.9 (0.2–4.6)	0.916	1.5 (0.2–9.3	0.683
30–39	1.5 (0.3–7.5)	0.625	1.6 (0.3–10.5)	0.610
40–49	4.0 (0.6–26.0)	0.146	2.4 (0.2–30.7)	0.490
Ever used modern contraceptive				
No	Ref		Ref	< 0.001
Yes	5.0 (2.9–8.6)	< 0.001	7.7 (3.4–17.5)	
Menstruated after delivery				0.003
No	Ref		Ref	
Yes	1.7 (1.0–2.9)	0.050	4.3 (1.7–11.3)	
Resumed sexual activity				0.011
No	Ref		Ref	
Yes	8.5 (3.7–19.3)	< 0.001	4.7 (1.4–15.4)	
Discussed family planning with partner				
No	Ref		Ref	0.044
Yes	9.4 (4.8–18.3)	< 0.001	3.1 (1.03–9.2)	
Partners’ approval of modern contraceptive use				
No	Ref		Ref	
Yes	6.0 (3.2–11.4)	< 0.001	18.1 (6.3–51.6)	< 0.001
I do not know	0.3 (0.1–0.8)	0.013	7.7 (1.6–38.1)	0.012
Family planning counselling at antenatal				
No	Ref		Ref	
Yes	2.4 (1.2–4.8)	0.009	3.5 (1.3–9.9)	0.016
Family planning counselling at postnatal				
No	Ref		Ref	0.139
Yes	3.4 (2.0–5.7)	< 0.001	2.0 (0.8–5.3)	
Knowledge of available family planning methods at the health facility				
No	ref		Ref	0.001
Yes	6.7 (3.8–11.8)	< 0.001	4.7 (1.9–11.5)	

## Discussion

This study sought to find the prevalence of modern contraceptive methods among postpartum women attending the child welfare clinic of Tema General Hospital and Tema polyclinic as well as assess the determinants of modern contraceptive use. Determinants of modern contraceptive uptake among postpartum women include past modern contraceptive use, resumption of sexual activity and menstruation, male partner involvement in contraception, family planning counselling during antenatal care and knowledge of the modern methods of contraception available at the health facility.

The prevalence of postpartum modern contraceptive use was 26.3% with the 95% confidence interval of 21.5 to 31.4%. This finding is in line with the modern contraceptive prevalence rate of 22% found during the GDHS of 2014 [12]. The low prevalence of modern contraceptive use among postpartum women in this study could result in rapid repeat pregnancies with the resultant short inter-birth interval, which have been shown by several studies [19–24] to be detrimental to the health of both the mother and the child. Past modern contraceptive use, return of menstrual cycle, resumption of sexual activity, discussion of family planning with male partner and male partners’ approval of contraceptives were found to be associated with postpartum modern contraceptive use. Other factors significantly associated with modern contraceptive use among postpartum women were family planning counselling during antenatal care and knowledge of at least one modern contraceptive method available at the health facility.

The most preferred method of contraception was injectables (29.8%) which was consistent with several studies around the world [1, 12, 25–27]. All these studies showed a preference of women for short acting hormonal contraceptive methods such as injectables and the pill. However, long acting reversible methods such as IUDs and implants as well as permanent methods are known to be more effective in pregnancy prevention [28]. In this study, the commonest reason given by women for not being on any modern contraceptive method was the fear of side effects (36.9%). Non-use of modern contraceptive methods due to health concerns and fear of side effects are also common reasons given by women in Africa and other parts of the world [12, 14, 29].

Past modern contraceptive use prior to the last pregnancy increased the likelihood of current modern contraceptive use among postpartum women. This association is also seen in other studies across the world [30, 31]. This relationship may be due to the fact that women recognize the benefits of modern contraceptive use after initial use and continue to use them later on in life. Perhaps, they realize that the myths and side effects of contraceptives are over-exaggerated when they use it themselves and continue to use them in the postpartum period to space and limit childbirth.

Additionally, women whose menstrual cycle had returned after delivery were more likely to use modern contraception. This findings is in consonance with existing evidence indicating that most women prefer to use contraception when they had resumed menstruation [25, 32]. A survey of 17 low and middle income countries [33] using secondary data from their demographic and health surveys revealed a trend of postpartum women waiting for the return of their menses before the uptake of a modern method of contraception. The public health implication of this finding is that it can lead to a rise in unplanned and unintended pregnancies because women mistakenly think postpartum amenorrhoea means they cannot become pregnant. Pregnancy is possible in postpartum women because ovulation, which is the releases of eggs by the ovary can occur before menstruation [34, 35].

As expected, women who had resumed sexual activity after delivery were more likely to use modern contraception. This finding is consistent with two studies from East Africa that found that resumption of sex predicts modern contraceptive use among postpartum women [32, 36].

It was also evident that family planning discussion among couples after last delivery increases postpartum contraceptive uptake. Hameed et al. [37] demonstrated in the Punjab Province of Pakistan, that when couples jointly make the decision to use contraceptives, its use was significant than when the decision was made to use it only by the woman. This goes a long way to show the importance of involving the male partner in decisions regarding contraception. Likewise, male partners’ approval of modern contraceptives increases its use. This association is not surprising because in many parts of the world especially SSA, husbands are the heads of families and major decisions such as fertility desires has to be approved by them.

Postpartum modern contraceptive use was significantly associated with family planning counselling during antenatal care as seen in several studies across the world [1, 30, 32, 38–41]. Appropriate family planning information and counselling at antenatal care visit allows women enough time to decide which method will be suitable for them in the postpartum period. Family planning counselling during antenatal care may motivate women who were otherwise indecisive to use modern contraceptive methods during the postpartum period.

Knowledge of at least one method of modern contraception available at the health facility increased modern contraceptive use by postpartum women. This relationship may be explained by the fact that in Ghana, health facilities are the major sources of some contraceptive methods [12] and therefore one is expected to know the available methods at the facility before choosing a suitable method.

The study is one of the few in the Tema area that seeks to determine the predictors of modern contraceptive use among postpartum women, thereby adding to the body of knowledge on postpartum contraception. Another strength of this study is that reliable data and appropriate methods were used, thereby ensuring that the findings reflect accurately on modern contraceptive use among postpartum women in the Tema area. A limitation of the study is in its design. The cross sectional study design made it impossible to establish causal relationships. Therefore, associations were the best ways to interpret results. Another limitation of the study was recall bias. Postpartum women had to recall certain reproductive health characteristics and behaviours that occurred in the past. Some of these behaviours were self-reported and there was practically no way to independently verify them. Sexual activity and contraceptive use are considered intimate issues in Ghana. This has a potential to lead to some reluctance in answering questions which bordered on those issues. However, this concern was addressed when the confidentiality and privacy of women were respected in the study. Again, this was a facility-based study in an urban setting, and therefore results can only be generalizable to similar urban settings.

## Conclusions

Postpartum modern contraceptive uptake is low among women in the Tema area. Injectables are the most preferred method of modern contraception while the fear of side effects is the major reason for modern contraceptive non-use. Modern contraceptive uptake among postpartum women in the Tema area is influenced by past modern contraceptive use, resumption of sexual activity and menstrual cycle after last delivery, the male partner’s involvement in contraception, family planning counselling given by health care providers during antenatal care and postpartum women knowing at least one methods of modern contraception available at the health facility.

There should be an increased advocacy by the Tema Metropolitan Health Directorate to engage men as partners in family planning educational programs since their influence is huge in modern contraceptive uptake among postpartum women. Efforts should be made at the facility level by authorities and health managers to strengthen family planning counselling during antenatal care.

## Data Availability

The dataset that supports the conclusions of this study is included in the article.

## Abbreviations

AOR: Adjusted Odds Ratio
CI: Confidence Interval
COR: Crude Odds Ratio
GDHS: Ghana Demographic and Health Survey
GHS: Ghana Health Service
IQR: Interquartile Range
SSA: Sub-Saharan Africa
TGH: Tema General Hospital
TP: Tema Polyclinic
WHO: World Health Organization.
